# Density Functional Study of Tetraphenylporphyrin Long-Range Exciton Coupling

**DOI:** 10.1002/open.201200020

**Published:** 2012-08-21

**Authors:** Barry Moore, Jochen Autschbach

**Affiliations:** a Department of Chemistry, University at Buffalo, State University of New York312 Natural Sciences Complex, Buffalo, NY 14260-3000 (USA) E-mail: jochena@buffalo.edu

**Keywords:** ab initio calculations, CD/LC/ORD, density-functional calculations, long-range exciton circular dichroism, porphyrins

## Abstract

The performance of time-dependent density functional theory (TDDFT) for calculations of long-range exciton circular dichroism (CD) is investigated. Tetraphenylporphyrin (TPP) is used as a representative of a class of strongly absorbing chromophores for which exciton CD with chromophore separations of 50 Å and even beyond has been observed experimentally. A dimer model for TPP is set up to reproduce long-range exciton CD previously observed for a brevetoxin derivative. The calculated CD intensity is consistent with TPP separations of over 40 Å. It is found that a hybrid functional with fully long-range corrected range-separated exchange performs best for full TDDFT calculations of the dimer. The range-separation parameter is optimally tuned for TPP, resulting in a good quality TPP absorption spectrum and small DFT delocalization error (measured by the curvature of the energy calculated as a function of fractional electron numbers). Calculated TDDFT data for the absorption spectra of TPP are also used as input for a ‘matrix method’ (MM) model of the exciton CD. For long-range exciton CD, comparison of MM spectra with full TDDFT CD spectra for the dimer shows that the matrix method is capable of producing very accurate results. A MM spectrum obtained from TPP absorption data calculated with the nonhybrid Becke88–Perdew86 (BP) functional is shown to match the experimental brevetoxin spectrum ‘best’, but for the wrong reasons.

## Introduction

The coupling of electronic excitations in molecular aggregates or in multi-chromophore molecules and metal complexes has long been an important research topic in chemistry and physics.^1^, ^2^ Of special interest are situations where the electric transition dipoles of the uncoupled excitations are geometrically arranged along a ‘helical’ path such that the coupled system exhibits electronic exciton circular dichroism (CD).^3^–^7^ Exciton CD reveals a great amount of information about the relative orientation of individual chromophores with respect to each other and about the distance between them. Exciton CD can be very strong, even if the individual chromophores have no intrinsic chirality. In leading order, and at large separation of the chromophores, the rotatory strengths of the coupled transitions (the integrated intensities of the CD of individual excitations) are determined simply by the lengths, relative orientations, and relative distances of the electric transition dipole vectors as well as by the energies of the uncoupled transitions.^8^–^10^ Equations for the ‘matrix method’ (MM) dipole coupling model^8^ are provided in the Discussion section to illustrate the case.

**Figure 1:**
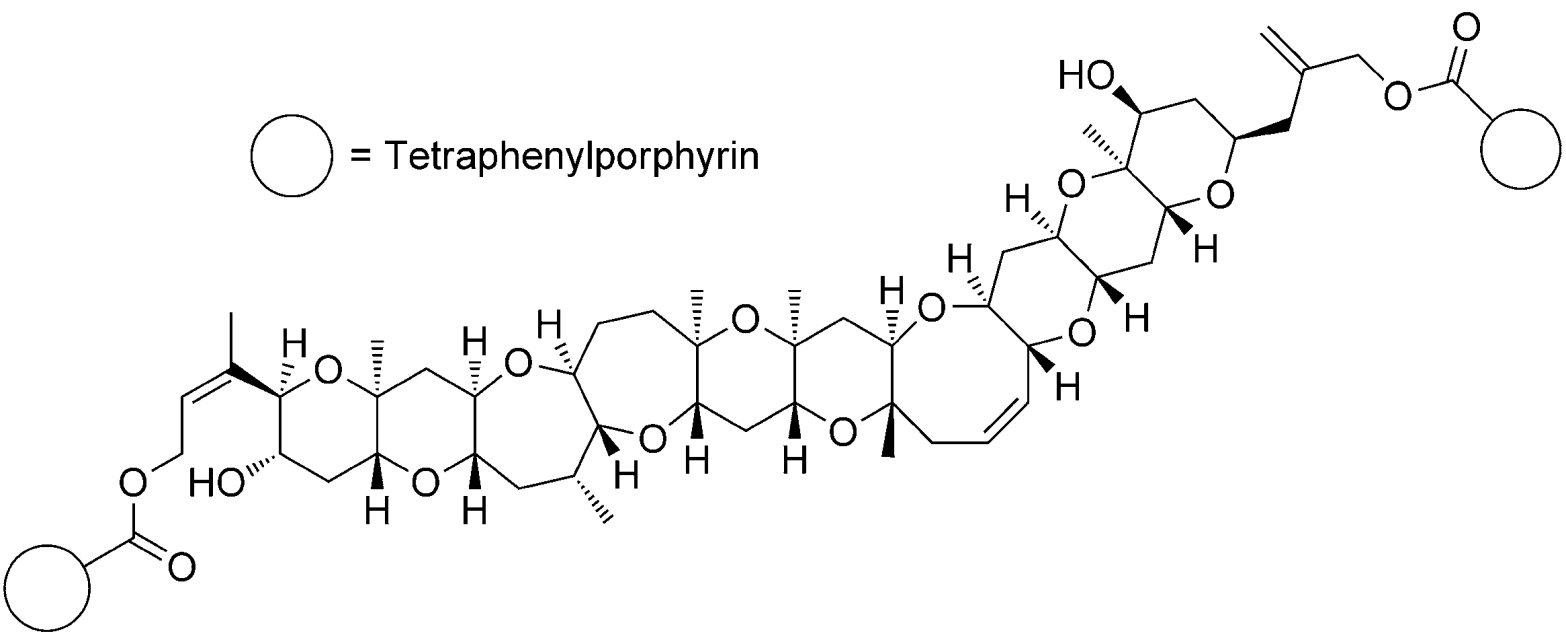
Brevetoxin substituted with TPP used in Ref. 11 to demonstrate long-range exciton CD.

Berova and collaborators have experimentally detected very long-range exciton CD with chromophore separations up to 50 Å for exciton coupling between two porphyrin based chromophores, for instance for tetraphenylporphyrin (TPP).^11^ Tsubaki et al. have reported observations of TPP exciton CD of substituted chiral oligonaphthalenes at even larger distances of approximately 66 Å.^12^, ^13^ The strong coupling between TPP substituents in suitably derivatized biomolecules has allowed researchers to derive important information about their three-dimensional structure. Examples where porphyrin-based exciton CD has been investigated or used in this context include the use of magnesium porphyrin to determine the absolute stereochemistry of chiral alcohols,^14^ a theoretical study of Soret band coupling of bis-porphyrin derivatives using various exciton coupling models,^15^ a determination of absolute stereochemistry of cyclic α-hydroxyketones with zinc–TPP tweezers,^16^ and investigations of the conformational space of DNA.^17^ A study of bis-porphyrin dimers of various derivatized biomolecules, including a brevetoxin bis-TPP derivative shown in Figure 1, has established the very long-range nature of the exciton CD.^11^

In first-principles calculations of electronic absorption and CD spectra, the size of the system—dictating the number of basis functions, *B*—is one of the major limiting factors because of the scaling of the computational effort with *B*. Presently, the most frequently utilized method for electronic spectra calculations is (linear response) time-dependent density functional theory (TDDFT).^18^–^20^ Reasonably efficient wave function-based methods for the treatment of exciton CD are available as well.^21^ The attractiveness of TDDFT derives from the fact that it incorporates electron correlation at a computational cost that is comparable to Hartree–Fock (HF) theory, if hybrid functionals are used for the exchange. The scaling is formally of order *B*^4^ but in practice often lower. Non-hybrid functionals afford better scaling yet. Still, the computational demand can be prohibitive if one attempts a reliable first-principles theoretical modeling of large systems that are of interest in bio-chemistry, not only because of the scaling as a function of *B* but also because of very large conformational spaces that may be encountered. The application of a dipole coupling model for well separated chromophores, or a more refined model including higher orders of the multipole expansion, offers a way out of this dilemma as far as exciton coupling is concerned.^15^ For instance, exciton coupling models have been applied successfully in a recent study of the CD spectrum of bacteriorhodopsin.^22^ Moreover, input data for such a model can be calculated from first principles for each chromophore separately, and the coupled excitations are then obtained from the lower-level coupling model at essentially negligible computational overhead. Herein, we explore this computational route for the long-range exciton CD of TPP pairs as encountered, for instance, in the aforementioned study of derivatized brevetoxin,^11^ in conjunction with the ‘matrix method’ (MM). One aim of this work is to investigate the performance of such a two-level computational model by comparing the results of a dipole-coupling scheme using TPP monomer TDDFT data as input with full TDDFT dimer calculations.

TDDFT with popular standard hybrid and non-hybrid functionals may afford large errors in calculated excitation energies, if the excitation has an explicit or hidden charge-transfer (CT) character.^23^–^27^ For multi-chromophore systems, the CT problem may also create large numbers of spurious low-energy CT excitations.^28^, ^29^ The CT problem of TDDFT can be corrected effectively by employing hybrid functionals with range-separated exchange,^30^–^35^, ^26^ in particular if the functional goes to pure HF exchange asymptotically (full long-range correction [LC]). However, the range-separation parameter in the exchange functional is strongly system dependent^36^ and should therefore be determined system-specifically. Recently, there have been ways proposed of how to achieve a system-specific optimal ‘tuning’ of the range-separation parameter as well as other parameters in the exchange functional in an ab initio sense, based on criteria rooted in density functional theory (DFT).^27^, ^37^–^43^ Another aim of this work is to investigate whether such a functional tuning is beneficial in the description of the TPP excitation spectrum and in the calculation of exciton coupling CD of TPP dimers. It is shown that the use of a system-specific optimally tuned range-separation parameter significantly improves the calculated TPP absorption spectrum compared with a universal parametrization. A LC functional is also shown to be the best choice for the study of long-range exciton coupling in full TDDFT calculations of the coupled system, as it suppresses spurious CT excitations. At large separations of the chromophores, it is shown that a simple dipole coupling model based on TDDFT monomer input data gives excellent agreement with long-range corrected TDDFT dimer spectra.

## Computational Details

A geometry optimization of tetraphenylporphyrin (TPP) was performed with the Amsterdam Density Functional (ADF) program,^44^–^46^ using the Becke88–Perdew86 (BP) functional,^47^–^50^ dispersion corrections as devised by Grimme et al.^51^ (see the ADF documentation regarding the DFT-D3 parametrization), and a combination of double-*ζ* polarized (C,N) and double-*ζ* (H) Slater-type basis sets from the ADF basis set library. Electronic absorption spectra of TPP were calculated with the NWChem program^52^ using time-dependent Hartree–Fock (TDHF)^53^, ^54^ and TDDFT,^55^–^57^ employing the 6-31G(d) Gaussian-type basis. The TDDFT calculations were carried out with the BP functional and with a fully long-range corrected hybrid functional with range-separated exchange based on the Perdew–Burke–Ernzerhof (PBE) exchange-correlation functional.^58^, ^35^, ^59^ We use the acronym LC-PBE0 for the range-separated functional in this work. Details about the optimal tuning of this functional are provided in the Results and Discussion section. The tuning procedure was followed by a series of single-point energy calculations for TPP with integer and fractional electron numbers using a fractional occupations/fractional total electron number code implemented in NWChem by one of the authors.^41^ For additional information on the functional tuning, please see Ref. 36 and our group’s previous work on functional tuning.^41^, ^40^ The basis sets were chosen because they are computationally efficient, but at the same time they produce acceptable results for the TPP monomer benchmark (see below). The excitation spectrum is dominated by valence transitions that do not mandate diffuse functions in the basis set.

A TPP dimer configuration was derived from a structure of the brevetoxin derivative BTX-D(1,1,42)-1,42-diol bis(*p*-[10′,15′,20′-triphenyl-5′-porphyrinyl] benzoate) (referred to as BTX-D) as shown in Figure 1 and chosen to model exciton coupling of TPP dimers at large spatial separations. The geometry was inferred from Ref. 11, where a CD spectrum for BTX-D has been previously reported. A CD couplet in the spectral range of the TPP Soret band has been assigned to exciton coupling of the respective TPP transitions at distances of up to 50 Å.^11^ A structure of BTX-D was obtained from a conformational search using Spartan′08 and subsequent molecular mechanics optimizations.^60^ The steroid linkage was then removed and each TPP unit was replaced with the BP optimized structures. The model affords an interchromophoric separation of approximately 42 Å. One of the TPP moieties was rotated along the centroid–centroid axis to match relative orientations as inferred from the experimental BTX-D CD spectrum.^11^ The resulting geometry is referred to as the TPP dimer in this article. Our aim was not to explore the full conformational space of BTX-D but rather to obtain a reasonable separation between the TPP moieties for a low-energy conformer (because of the strong distance dependence of the exciton CD) in order to benchmark the matrix method (MM) results in comparison with full TDDFT calculations for the dimer. MM dipole-coupled CD spectra for the dimer were based on monomer spectral data calculated with TDHF, BP, and a global and tuned parametrization of LC-PBE0. Full TDDFT dimer calculations of CD spectra were performed with NWChem and Gaussian^61^ with the 6-31G(d) basis. Dimer TDDFT calculations with the BP functional were performed with Gaussian due to convergence problems with the NWChem TDDFT code for large numbers of excitations (which are needed with BP in order to cover the experimentally accessible spectral range). Most of the CD spectra are based on the dipole-length representation of the rotatory strength. One of us has recently reported a TDDFT implementation in NWChem for CD spectra calculations with gauge-including atomic orbitals (GIAOs, or London orbitals) and with the dipole-velocity gauge.^62^ This code was used to test that the dimer spectra reported herein are not contaminated by a gauge-origin dependence (see below and Ref. 62). For more information on the MM, see work by Schellman et al.^8^ and previous work by our group on exciton coupled CD spectra.^10^ All calculated absorption and CD spectra were Gaussian broadened with a *σ* value of 0.13 eV.

## Results and Discussion

The geometry optimization of tetraphenylporphyrin (TPP) yielded a porphin backbone adopting a saddleback-shaped geometry and phenyl substituents with dihedral angles of approximately 66 ° between the porphin plane and the phenyl planes. The geometry is consistent with previous theoretical results^63^–^65^ and with experimental data.^66^ The remainder of this section is divided into three parts: 1) *γ*-tuning of the LC-PBE0 functional for TPP; 2) an analysis of the TPP absorption spectrum calculated with various functionals, and comparisons with previously published calculated spectra and with experiment; 3) the analysis of the exciton CD for the TPP dimer model.

### Tuning of the LC-PBE0 functional for TPP

A system-specific tuning of the LC-PBE0 functional for TPP was carried out by minimizing *J*′^2^, of Equation (1), as a function of the range-separation parameter *γ* in the exchange functional. Here, *N* is the number of electrons for neutral TPP.


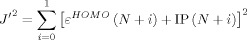
(1)

Specifically, we used the following range-separation of 1/*r*_12_ in the exchange [Equation (2)]:^32^


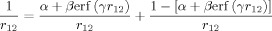
(2)

The ionization potential (IP) is calculated as the difference of total energies, IP(*N*)=*E*(*N*−1)−*E*(*N*), and similarly for the corresponding (*N*+1)-electron system. Further, *ε*^HOMO^ is the energy of the highest occupied molecular orbital (HOMO). For the elusive exact universal density functional, *J*′^2^=0, the negative of the HOMO orbital energy would correspond exactly to the first ionization potential (IP). For an approximate functional, the idea is to determine the range-separation parameter such that an optimally small *J*′^2^ is obtained, thereby giving physical meaning to the HOMO energy of both the *N*-electron and the (*N*+1)-electron system. The process is thought to improve the fundamental gap.^42^ The other two parameters in Equation (2), *α*=0.25 and *β*=0.75, were kept as in the originally proposed parametrization,^35^ which affords 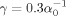. We noted in previous work that tuning according to the IP criterion requires *α*+*β*=1.^40^, ^41^ For a procedure to determine *α* to minimize the DFT delocalization error simultaneously with an optimal tuning of *γ* per Equation (1), see Ref. 40. The DFT delocalization error^67^ can be quantified by the behavior of *E*(*N*) for fractional and integer electron numbers *N*. The exact energy should afford linear segments between integers, with slopes changing discontinuously^68^ at integer values of *N*. Curvature in plots of *E*(*N*) is therefore indicative of delocalization error.

**Figure 2:**
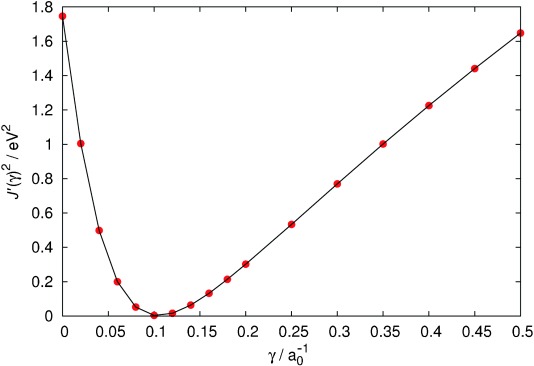
*J*′^2^ of Equation (1) versus *γ* for TPP, LC-PBE0 functional (*α*=0.25, *β*=0.75). The optimal value determined from an interpolating function is 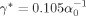.

In Figure 2, *J*′^2^ calculated for TPP is shown for varying *γ*. Recent theoretical studies have shown^27^, ^41^ that as the size of a conjugated delocalized π system increases, the optimal range-separation parameter *γ* tends to decrease. In turn, *γ* is the reciprocal of a cut-off distance beyond which HF exchange in the functional starts to dominate. That is, a reduced value of *γ* indicates that the delocalized π system benefits from local DFT exchange acting over a longer range of interelectronic distances. The optimal γ^*^ value for TPP is 0.105 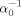, that is, much below the originally proposed value of 0.3 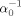.

**Figure 3:**
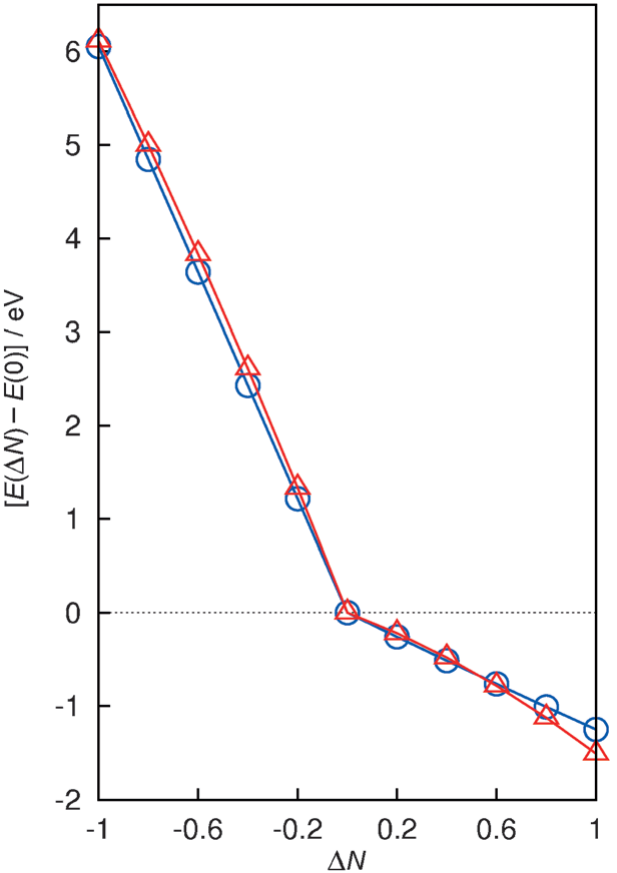
*E*(Δ*N*) of TPP calculated for standard LC-PBE0 (▵) and the optimally tuned LC-PBE0 (○) functional. Δ*N* is the difference between the electron number of the calculated system and *N* of neutral TPP. The curvature measures for the Δ*N*<0 (electron deficient) and Δ*N*>0 (electron rich) regimes in the form of the (Δ*N*)^2^ coefficients of quadratic fits of *E*(*N*) are LC-PBE0 (−0.69, −0.51), LC-PBE0-γ^*^ (−0.04, 0.06).

The behavior of *E*(*N*) as a function of a fractional electron number *N* is compared, in Figure 3, for the original parametrization of LC-PBE0 (*α*=0.25, *β*=0.75, *γ*=0.3) and the tuned version (γ^*^=0.105). We refer to the tuned functional as LC-PBE0-γ^*^ from here on.

The stock parametrization of LC-PBE0 affords delocalization error for TPP, as demonstrated by the curvature in *E*(*N*). The negative curvature indicates that, for TPP, the value *γ*=0.3 gives too little delocalization. The behavior of *E*(*N*) for the tuned functional is much better. The curvature is nearly vanishing for both the electron-deficient and the electron-rich species. Because of the reasonably small curvatures obtained with γ^*^ at *α*=0.25, we decided to forego a simultaneous optimization of *α* and *γ* for TPP.

### TPP excitation spectrum

**Figure 4:**
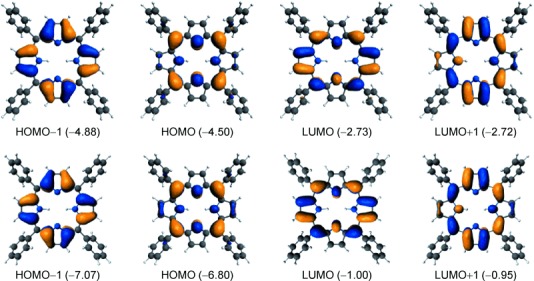
Frontier orbitals (HOMO−1, HOMO, LUMO, and LUMO+1 respectively) for TPP calculated with BP (top) and LC-PBE0 (bottom). Orbital energies [eV] are given in parentheses. Orbital isosurfaces for ±0.03 a.u.

Since the excitation spectrum of TPP in this work is used as the input for the matrix method (MM) coupling model, it is important to assess the quality of the spectrum calculated at various levels of theory. The currently accepted assignment of the experimental TPP spectrum, in reference to the Gouterman model,^69^ is as follows: Weak absorption bands (Q-bands) at 1.86 eV (Q_*x*_) and 2.26 eV (Q_*y*_) are assigned to HOMO-to-LUMO and HOMO-to-LUMO+1 transitions, respectively, in a molecular orbital (MO) representation. Isosurfaces of the relevant MOs are shown in Figure 4. A very intense absorption band around 3.06 eV, commonly referred to as the Soret band, is caused by a pair of transitions (B_*x*_, B_*y*_) that are assigned to HOMO−1-to-LUMO and HOMO−1-to-LUMO+1, respectively. The pairs of excitations responsible for the Q and B bands are calculated as ^1^B_3*u*_/^1^B_2*u*_ pairs. The symmetry labels for the excitations refer to the *D*_2*h*_ point group, even though the symmetry of the optimized TPP geometry is lower (*C*_2*v*_). However, the MO contributions to the excitations and the MO nodal patterns are similar to porphin as described by the Gouterman model. Note that the N transitions, assigned in previous and current works, are not considered in the Gouterman model, and therefore alternate criteria were taken from the literature. An excitation around 3.5 eV (N_*x*_) has been assigned to a ^1^B_3*u*_ excited state in Ref. 70 based on similarity-transformed equation-of-motion coupled-cluster singles and doubles (STEOM-CCSD) calculations on free base porphin. From Ref. 63, the N_*y*_ transition was assigned to the corresponding porphin ^1^B_2*u*_ excitation with dominant MO contributions from HOMO−3 to LUMO+1 which is consistent with the calculations in this article. The spectral features of TPP above 4 eV are ignored in this work since the Q, B, and N transitions are the ones most important for the experimentally observed exciton coupling CD of TPP dimers.

In Table 1, the Q, B, and N transitions of TPP calculated by TDHF, and by TDDFT with various functionals, are compared with previously reported calculated^63^ and with experimental data.^71^ The transition assignments were made based on the dominant contributions from occupied (occ) and unoccupied (unocc) MOs to the excitation transition densities, and in comparison with previous porphin^70^, ^72^ and TPP^63^ calculations as well as the Gouterman model. The frontier orbitals obtained with Becke88–Perdew86 (BP) and LC-PBE0 are compared in Figure 4. Differences between the MOs obtained with different functionals are hardly visible in the isosurface plots. The nodal patterns of the frontier orbitals match those of the Gouterman model^69^ and agree with previous DFT calculations.^63^, ^73^

**Table 1:** Computed excitation energies Δ*E* [eV] and oscillator strengths *f* for the Q, B, and N transitions of TPP

	TDHF	BP	LC-PBE0	Tuned LC-PBE0	PBE0[a]	CAM-B3LYP[a]	M05-2X[a]	Exp.[b]
Q_*x*_(^1^B_3*u*_)	1.63	2.02	1.91	2.16	2.21	2.11	2.22	1.86
*f*	0.00	0.03	0.01	0.02	0.04	0.02	0.02	
Δ*E*	−0.23	0.16	0.05	0.30	0.35	−0.25	0.36	
Q_*y*_(^1^B_2*u*_)	1.78	2.14	2.18	2.34	2.36	2.31	2.41	2.26
*f*	0.00	0.05	0.03	0.05	0.05	0.03	0.04	
Δ*E*	−0.48	−0.12	−0.08	0.08	0.10	0.05	0.15	
B_*x*_(^1^B_3*u*_)	3.71	3.13	3.47	3.28	3.18	3.28	3.32	3.06
*f*	1.77	0.89	1.44	1.07	1.43	1.68	1.75	
Δ*E*	0.65	0.07	0.41	0.22	0.12	0.22	0.26	
B_*y*_(^1^B_2*u*_)	3.77	3.09	3.54	3.39	3.33	3.33	3.36	
*f*	2.07	1.05	1.74	1.52	1.73	1.92	1.94	
Δ*E*	0.71	0.03	0.48	0.33	0.27	0.27	0.30	
N_*x*_(^1^B_3*u*_)	4.85	3.73[*]	4.34	3.82	3.67	4.08	4.13	*∼*3.5
*f*	0.63	0.18	0.55	0.64	0.39	0.45	0.39	
Δ*E*	1.35	0.23	0.84	0.32	0.17	0.58	0.63	
N_*y*_(^1^B_2*u*_)	5.57	3.75[*]	4.80	3.93	3.78	4.43	4.48	
*f*	0.07	0.17	0.02	0.09	0.05	0.05	0.04	
Δ*E*	2.07	0.25	1.3	0.43	0.28	0.93	0.98	

[a] PCM/TDDFT calculations on PCM/PBE0/6-31G(d) TPP geometries.^63^

[b] TPP gas phase spectrum (position of band maximum).^71^ Δ*E*=*E*(calculated vertical transition)−*E*(experimental band maximum).

[*] Assignment based on energy. See text for details.

**Figure 5:**
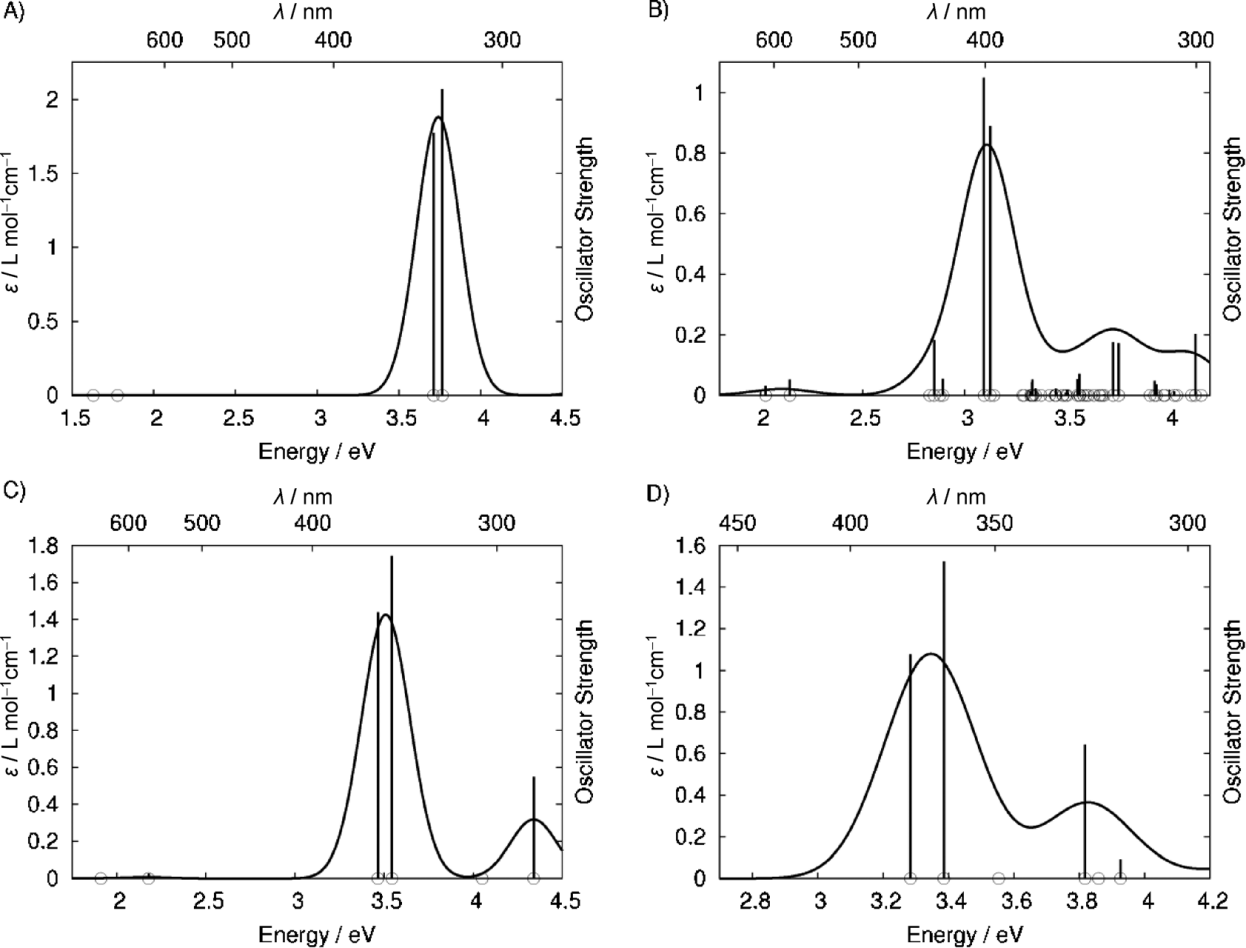
Calculated ‘stick spectra’ (excitation energies indicated by circles on the abscissa, oscillator strengths plotted as sticks), and Gaussian-broadened absorption intensities of TPP for A) TDHF; B) BP; C) LC-PBE0; D) LC-PBE0-γ^*^ (tuned). Note that all spectra in this work are plotted on an energy scale increasing from left to right. That is, the wavelength increases from right to left, as shown in the wavelength scale at the top of each subfigure.

Simulated spectra are shown in Figure 5. The THDF calculation yields Q bands which are red shifted with respect to the experiment, while the B and N bands are blue shifted. The assignment of the transitions, based on dominant contributions to the transition density matrix from occupied and unoccupied MOs, is in qualitative agreement with the Gouterman model. The N bands can be assigned as transitions from HOMO−4 to LUMO (N_*x*_) and HOMO−4 to LUMO+1 (N_*y*_) where HOMO−4 of the HF calculation is equivalent to HOMO−3 for the DFT calculation of Ref. 63 and the present calculations for BP, LC-PBE0, and tuned LC-PBE0.

The BP functional yields blue shifted Q_*x*_ and red shifted Q_*y*_ transitions, with an assignment of these excitations that is in agreement with the Gouterman model. According to this model and the accepted assignment of the spectrum, the B_*x*_ excitation should be lower in energy than B_*y*_. The BP functional incorrectly reverses the energies. This finding is consistent with a BP spectrum previously reported in Ref. 74. An apparent N band may be attributed to a modestly intense pair of transitions at 3.7 eV, but the excitations do not have the expected MO contributions. With the BP functional, the excitations with strong HOMO−3-to-LUMO and HOMO−3-to-LUMO+1 character expected for the N transitions appear at lower energy than the Soret peak, that is, below 3 eV. Moreover, the BP ‘stick spectrum’ in Figure 5 reveals many spurious excitations, in particular between 3 and 4 eV, with (mostly) low intensity. These transitions are not seen in the other spectra obtained with HF and asymptotically correct density functionals. The occurrence of these spurious transitions is likely a consequence of the incorrect long-range behavior of the BP exchange-correlation (XC) potential. In recent work, Baer, Kronik et al. noted that ‘charge-transfer-like’ excitations may occur in extended π chromophores, which render calculations with functionals that do not afford the correct long-range behavior suspicious.^39^ The XC potentials of TDHF and LC-PBE0 (standard and tuned version) afford the correct long-range behavior, and therefore spurious transitions are not as prevalent. Clearly, the BP spectrum is seriously deficient despite the fact that the broadened absorption intensity resembles the experimental spectrum reasonably well.

The LC-PBE0 and LC-PBE0-γ^*^ functionals predict blue shifted Q, B, and N bands when compared to experiment (with the exception of Q_*y*_ calculated with LC-PBE0). The tuning procedure is designed to improve the HOMO–LUMO energy difference toward the fundamental gap, and it has been shown that TDDFT response calculations based on the resulting functionals provide improved excitation energies as well^42^ (where, similar to HF, the optical gap is calculated to be smaller than the HOMO–LUMO gap). From Table 1, the Q_*x*_ transition is seen to be predicted worse for the tuned functional than for the stock parametrization. For all other transitions, however, the tuned LC-PBE0-γ^*^ functional gives excitation energies that are closer to the experimental band maxima. The *γ* tuning also closes the gap between the B and N bands, which is favorable.

**Figure 6:**
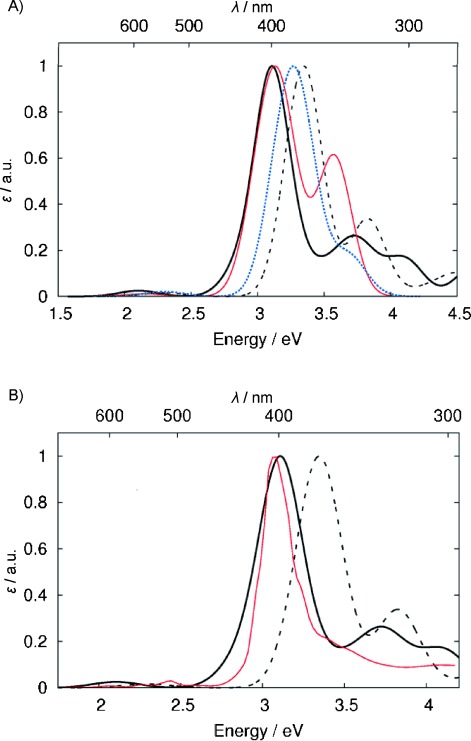
A) Calculated absorption spectra compared to previously reported theoretical absorption spectra:^63^, ^73^ BP (—), tuned LC-PBE0 (- - - -), GW+BSE (—), PBE0 (- - - -). B) Selected calculated spectra compared to experiment:^71^ BP (—), tuned LC-PBE0 (- - - -), experiment (—). The intensities were scaled such that the Soret peaks have the same intensities.

In Figure 6, the BP and tuned LC-PBE0 absorption spectra are compared to calculated spectra reported previously in the literature (GW+BSE^73^ and TDDFT with the PBE0 functional^63^) and to an experimental spectrum.^71^ The TDHF spectrum is excluded because the excitation energies do not agree well with experiment. LC-PBE0 is also excluded because the tuned version yields an overall better spectrum. The GW+BSE and PBE0 spectra were generated by Gaussian line broadening (*σ*=0.13) based on excitation data taken from Ref. 73 and Ref. 63, respectively. For better comparison, the Soret maxima were scaled to the same value and the spectra are given in arbitrary units (a.u.).

The BP density functional is computationally rather inexpensive. However, as pointed out above, despite the apparent reasonable agreement with experiment, the deficiencies of the functional are clear when considering the assignments of the relevant transitions. For instance, the reader is reminded that the ‘N’ band in the BP spectrum is not the N-band proper. The computational savings from not including exact exchange (and a range separation of the exchange) are also not as advantageous as one might think because of the need to calculate a large number of excitations to cover the spectral range up to 4 eV (most of which are unphysical). This problem is exacerbated in the dimer calculations discussed below. The tuned LC-PBE0-γ^*^ functional performs well in comparison. We also note good agreement of the tuned hybrid with the GW+BSE calculation, apart from a modest blue shift.

### Dimer calculations: TDHF, TDDFT, and the dipole coupling model

**Figure 7:**
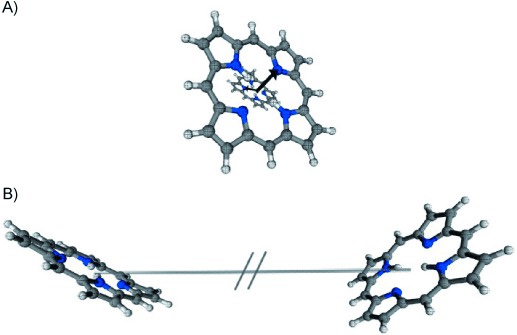
A) Perspective image of the TPP dimer model with an interchromophoric distance of 42 Å. B_*x*_ transition dipole moment vectors are indicated by arrows (dihedral angle: −118 °). B) Side-view of the dimer, showing the correspondence to the setup in Figure 11. Phenyl groups were replaced with hydrogen for clarity, and interchromphoric distance was reduced for the plot.

A TPP dimer was investigated in a relative arrangement approximating the geometry of BTX-D, as described in the computational details. The dimer model along with the relative orientation of two selected transition moments (B_*x*_) is shown in Figure 7. This TPP dimer was used for all calculations discussed in this subsection.

Given the large separation of the chromophores, it is important to investigate if the CD spectra calculated for the full dimer are affected by the gauge-origin problem of magnetic properties in finite-basis set calculations (see Ref. 62 and Ref. 75 for a detailed discussion of the problem in the context of CD spectra calculations, and for additional literature devoted to this topic).

**Figure 8:**
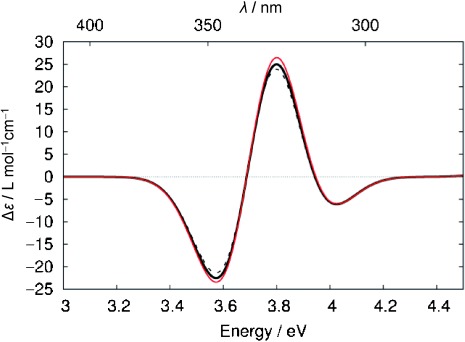
TDHF spectra of the TPP dimer shown in Figure 7, calculated with dipole-length (—), dipole-velocity (- - - -), and GIAO dipole-length (—) rotatory strengths.

Figure 8 shows a comparison of TDHF calculations of the full dimer CD spectrum based on different rotatory-strength representations: dipole-length (standard basis), dipole-velocity, and dipole-length with use of gauge-including atomic orbital (GIAO) basis functions. The velocity and GIAO rotatory strengths are both origin invariant by design, but the velocity-gauge may be affected more strongly by basis-set incompleteness. As the calculated CD spectra demonstrate, there is virtually no difference between the three CD spectra. Consequently, in order to save computational resources, all other dimer calculations have been performed with the dipole-length gauge without GIAOs.

In the matrix method (MM) coupling model, the individual excitations in individual chromophores are treated as quasi-particles (excitons) that interact electrostatically via their electric and magnetic multipole moments. For details and additional citations to original references not cited herein please see the Appendix of our previous work.^10^ A coupled dimer with a single transition per monomer, for instance, is described by the Hamiltonian [Equation (3)],


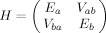
(3)

where the monomer excitations are labeled *a*, *b*, etc. and have energies *E_a_*, *E_b_*, etc. To lowest order in the multipole expansion, the coupling only considers the electric transition dipole moment vectors ***d**_a_*, ***d**_b_*, etc., which gives rise to the electrostatic interaction [Equation (4)]:


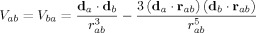
(4)

Here, ***r**_ab_*=***r**_b_*−***r**_a_* is the distance vector between the chromophore centers ***r**_a_* and ***r**_b_* (we use the center of nuclear charge), and *r_ab_* its length. Electrostatic units are used, where the square of an electric dipole divided by a volume yields an energy, and a magnetic dipole has the same unit as the electric dipole. In SI units, the equation for the potential carries an additional factor of (4π*ε*_0_)^−1^. In the MM, the magnetic transition dipoles associated with the uncoupled excitations are obtained from the electric transition dipoles via Equation (5).


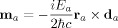
(5)

The excitation energies of the coupled system are the eigenvalues of *H*. To facilitate the next step, the eigenvectors of *H* are collected column-wise in a matrix *C*, and the uncoupled electric and magnetic transition dipoles are collected in matrices ***D***^0^ and ***M***^0^, respectively. The coupled transition moments ***d**_i_*, ***m**_i_* for excitations *i*=1, 2, etc. in the compound system are then obtained as the columns of the matrix products ***D***^0^*C* and ***M***^0^*C*. The rotatory strength of each coupled transition *i* is given as 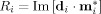 and the dipole strength as 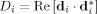. In the MM spectra reported in this subsection, the transition dipoles from a number *M* calculated excitations for the TPP monomer (using *M*=25 for TDHF, LC-PBE0, and LC-PBE0-γ^*^, and *M*=100 for BP) have been used in the coupling model. That is, dipole interactions between pairs of transition dipoles centered on different TPP moieties were calculated and used in a *2 M* by *2 M* matrix model in order to obtain the coupled excitation energies and rotatory strengths for the dimer.

**Figure 9:**
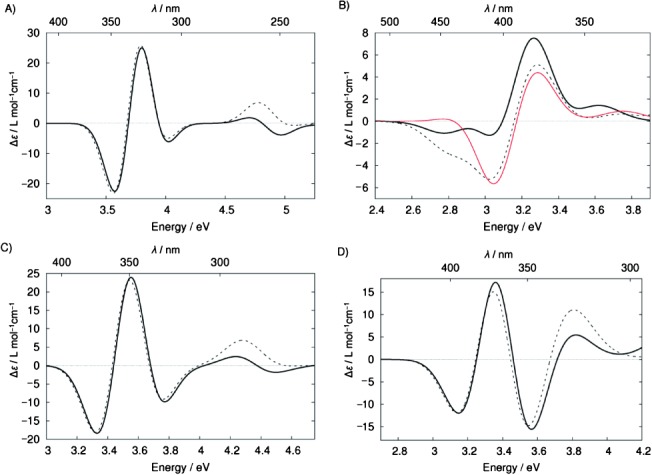
TPP dimer CD spectrum. Corresponding MM (- - - -) versus full calculation (—) for A) TDHF, B) BP, C) LC-PBE0, and D) LC-PBE0-γ^*^. Note: B) contains an additional MM spectrum (—) leaving out spurious transitions (see Results and Discussion section).

Figure 9 displays the broadened CD spectra of the TPP dimer of Figure 7 obtained using the matrix method with calculated TPP monomer spectral data, and from full dimer calculations. The MM spectra generated from monomer data for a particular functional are indicated by dashed lines. For the theoretical methods that afford a correct long-range behavior of the XC potential (TDHF, and TDDFT with LC functionals), the agreement with the full dimer spectra is excellent. Some deviations occur near the high-energy cutoff of the spectra. We consider this an artifact of the upper-energy cutoff for the TDDFT dimer spectra, made necessary by the memory limitations of the code used. That is, the upper energy range of the broadened dimer spectra may change in intensity if additional, possibly intense, excitations were available to generate the broadened spectra. For the exciton CD in the energy range of the calculated TPP Soret bands, the excellent agreement of the MM spectra with the full TDDFT spectra (HF and LC functionals) demonstrates that this couplet is indeed attributable to long-range dipole interactions of the intense TPP B transitions, along with some dipole-coupling contributions from the Q and N transitions.

The BP dimer spectrum is clearly affected strongly by the TDDFT CT problem. The dimer calculation afforded a large number of spurious transitions; over 150 excitations were needed to cover the rather small energy range shown in Figure 9. For comparison, 100 excitations in the BP monomer spectrum, many of which are already spurious, reach up to 5.1 eV. Some of the deficiencies noted for the TPP monomer spectrum are not hidden anymore in the dimer. A MM CD spectrum constructed from the Q, B, and higher energy (‘N′) transitions of the BP calculation (Figure 9 B) leaves out spurious transitions and shows that such a coupling would at least yield a qualitatively correct spectrum. In contrast, at 42 Å separation of the TPP moieties, the LC-PBE0 calculations (standard and tuned versions) afford the expected physically correct picture: The exciton CD of the dimer just above 3 eV is caused by simple electrostatic coupling, mainly, of the TPP B transition dipoles.

The deficiencies in the BP dimer TDDFT CD spectrum therefore have several origins. For instance, the MM(BP) spectrum displays a low-energy tail of the first (negative) CD band that is not visible in the dimer calculations with the other functionals. As pointed out above, the excitations that would be expected to form the TPP N band are obtained as pre-Soret features in the BP monomer spectrum (see also Ref. 74). It is the dipole coupling of these excitations that creates the intense low-energy tail in the MM(BP) CD spectrum. The full dimer TDDFT spectrum, however, is not even in agreement with the dipole coupling model based on deficient BP monomer data. The charge-transfer problem/wrong asymptotic behavior of the XC potential creates many additional spurious excitations, along with exciton CD from coupling among these and more physical transitions. These problems render the BP dimer CD spectrum altogether unreliable. For truly long-range coupling of excitations, it is possible that any ‘pure’ functional component with a wrong asymptotic behavior causes problems akin to those found for BP. This would also affect popular functionals such as B3LYP or PBE0 which afford only 20 and 25 % HF exchange, globally, in the exchange part of the functional. In comparison, the LC hybrid functionals appear unproblematic, as expected.

**Figure 10:**
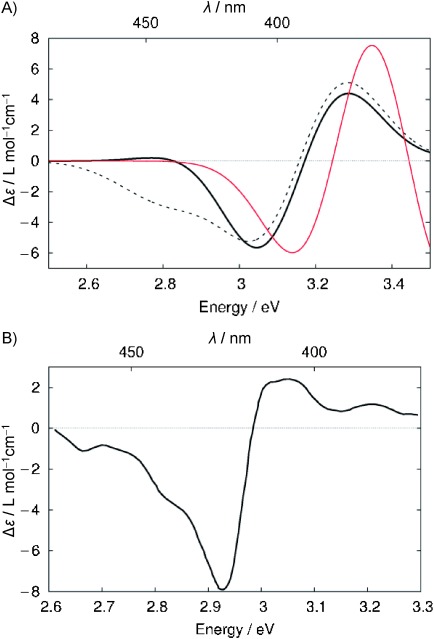
A) Selected MM spectra for the TPP dimer shown in Figure 7: MM(BP)-QBN (—), MM(BP)-Full (- - - -), MM(LC-PBE0-γ^*^) (—). The intensity has been divided by 2 for MM(LC-PBE0-γ^*^). B) Experimental spectrum of BTX-D (—) in MeOH/H_2_O (4:1) with addition of 0.1 μm CsCl; spectral data are taken from Ref. 11 and converted from a wavelength to an energy scale (see caption of Figure 5).

Figure 10 displays MM spectra obtained from BP and LC-PBE0-γ^*^ next to an experimental CD spectrum for BTX-D (see Computational Details). Based on the intensity of the simulated CD spectra, considering that computed intensities may typically deviate from experiment by factors of two, the magnitude of the BTX-D circular dichroism is consistent with exciton coupling of the TPP Soret (B) transitions at distances of 40–50 Å. Additional features seen in the experimental BTX-D spectrum but not in the MM(LC-PBE0-γ^*^) spectrum may be potentially attributed to excitations in the steroid backbone, vibronic fine structure of the excitations, the conformational flexibility of BTX-D, and possibly interactions of BTX-D with the solvent. The calculated spectra have been broadened empirically, with a uniform Gaussian width independent of the excitation energy. The full TDDFT dimer spectra in Figure 9 as well as the MM spectra indicate that the exciton couplet from the Soret bands can be nonconservative, for instance due to coupling of additional transitions with the B transitions. Moreover, if the broadening of the spectrum increases significantly at higher energies (shorter wavelengths), the observed trough and peak heights of the exciton CD couplet would differ. It is possible that such mechanisms lead to the much weaker observed CD intensity above 3 eV (below about 420 nm) in the experimental spectrum, compared to the negative CD band between 2.9 and 3 eV. However, other factors cannot be ruled out at this point. A full modeling of the spectrum would require exploration of the conformational space of BTX-D along with solvent effects, which is beyond the scope of this work. We note in passing that force-field based dynamics and conformer searches may bias calculations toward certain configurations.^76^ Careful benchmarks, possibly in comparison to ab initio molecular dynamics, appear to be in order.

An interesting point arising from the comparison in Figure 10 is the apparently ‘best’ agreement of the MM(BP) spectrum with the experimental CD of BTX-D. Based on the analysis presented above, the low-energy tail in the MM(BP) spectrum is generated by exciton coupling of spurious pre-Soret N-type transitions in the monomer. Therefore, this calculated MM(BP) spectrum looks more similar to experiment than the other dimer spectra presented in this section for entirely wrong reasons. More accurate calculations with LC functionals expose this problem.

**Figure 11:**
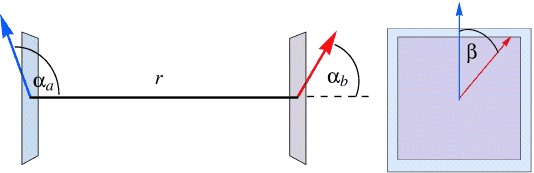
Dipole-coupling setup for two transitions, as discussed in the text. The angles are counted positive as drawn. The planes are perpendicular to *r_ab_*.

In the remainder of this section we consider additional aspects of the dimer exciton CD that can be extracted from the dipole coupling model. To facilitate the discussion, consider first a simplified MM setup with two degenerate uncoupled transitions, *E_a_*=*E_b_*=*E*, at a distance *r*, with identical electric transition dipole magnitudes *d_a_*=*d_b_*=*d*. For a situation where the two dipole vectors form angles of *α_a_* and *α_b_*, respectively, with *r_ab_* as shown in Figure 11, and adopt a dihedral angle of *β* along *r_ab_*, the potential *V_ab_*=*V_ba_*=*V* works out to be as given by Equation (6).


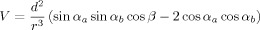
(6)

The excitation energies for the coupled system are *E*_*1/2*_=*E*±*V*. The corresponding rotatory and dipole strengths are given by Equations (7 a) and (7 b).


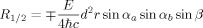
(7a)


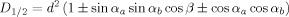
(7b)

As expected, the rotatory strengths change sign as the dihedral angle changes sign because of the sin *β* term. The sin *α*_*a/b*_ terms also indicate that the rotatory strengths vanish if one of the dipoles points along the inter-chromophore vector.

The CD couplets seen experimentally for TPP dimers have been assigned to coupling between the intense Soret transitions, which is consistent with our dimer calculations. The transition dipole vectors for the B transitions lie in the porphin plane. Assume a parallel TPP stack, that is *α_a_*=*α_b_*=90 °. If only two transitions were coupled, one *B_x_* or *B_y_* from each monomer, the dihedral angle adopted in the dimer model of Figure 7 would produce a couplet with the opposite sign of the full TDDFT or MM spectra, and opposite to experiment.

A MM coupling model for both monomer transitions, B_*x*_ and B_*y*_, results in a 4×4 Hamiltonian matrix. As additional data demonstrate (see the Supporting Information), for a ‘stacked-disk’ arrangement, the exciton CD vanishes if the monomer transitions are degenerate or nearly degenerate. As long as the excitations are degenerate, the CD couplet is negligible in a one-side-tilted stacked-disk arrangement (*α_a_*=90 ° and *α_b_*≠90 °). A substantially stronger exciton CD is created for relative monomer orientations with both *α* angles deviating substantially from 90 ° and, optionally, a substantial energetic splitting between the B_*x*_ and B_*y*_ transitions of the monomer. These conditions are fulfilled for the dimer model shown in Figure 7. Therefore, the single TPP dimer conformer used to represent an approximate average BTX-D structure affords a physically reasonable setup, geometrically and electronically as far as the TPP moieties are concerned, for a study of the long-range exciton CD of porphyrin-based chromophore dimers.

## Conclusions

Tetraphenylporphyrin (TPP) has been used in this work as a representative for a strongly absorbing chromophore for which measurements of long-range exciton circular dichroism (CD; at interchromophore distances of up to 50 Å) have been previously reported. Two main questions were addressed: 1) How accurate is an exciton coupling model such as the matrix method (MM) with input from time-dependent density functional theory (TDDFT) for the description of exciton CD at such spatial chromophore separations? 2) What types of density functionals are best suited for full TDDFT calculations of long-range exciton CD, and what functionals are best suited to generate reliable input data for a lower-level model such as the MM? In the context of these questions, an ‘optimal tuning’ of hybrid functionals with range-separated exchange has been addressed as well.

The results were found to be unambiguous: For long-range exciton coupling of large π chromophores, the use of a fully long-range corrected hybrid functional with range-separated exchange is beneficial for both 1) and 2). When reliable input data are generated from first principles methods, the matrix method based on a simple electric-dipole coupling is seen to produce good-quality exciton CD spectra for TPP dimers at spatial separations inferred from experimental data. For chromophores with extended π systems, it appears to be highly beneficial to optimally tune the range-separation parameter, as it is strongly system-specific. For the TPP test case, the tuning produced a much smaller range-separation parameter than typically used in global parametrizations of range-separated hybrids. The tuning resulted in a more accurate absorption spectrum, and significantly reduced DFT delocalization error as evidenced by small curvatures of *E*(*N*) when plotted for electron numbers (*N*) around the neutral system. These findings are in line with recent work by Baer, Kronik, et al. on the excitations of extended π systems^39^, ^42^ and recent findings by our group on the performance of range-separated hybrids in calculations of the optical rotation of helicenes.^41^ The disastrous dimer TDDFT results obtained with the ‘pure’ functional BP demonstrate that systems with spatially separated chromophores potentially pose problems for all functionals that retain a fraction of approximate local DFT exchange asymptotically. Even a MM spectrum based on BP data for the TPP monomer was found to be strongly deficient, due to exciton coupling in the dimer of spurious TPP N bands appearing energetically below the B transitions. Curiously, this spectrum agrees ‘best’ with an experimental TPP exciton coupling CD spectrum reported in Ref. 11 for a brevetoxin derivative.

## Supplementary material

Detailed facts of importance to specialist readers are published as ”Supporting Information”. Such documents are peer-reviewed, but not copy-edited or typeset. They are made available as submitted by the authors.
