# Supplementary material for: Density Functional Study of Tetraphenylporphyrin Long-Range Exciton Coupling
Source: ChemistryOpen. 2012 Aug 21;1(4):184–94. doi: 10.1002/open.201200020 (PMC3922448; doi:10.1002/open.201200020)
Supplement: Supplementary file 1 [file open0001-0184-SD1.pdf]

ChemistryOPEN

Supporting Information

© Copyright Wiley-VCH Verlag GmbH & Co. KGaA, 69451 Weinheim, 2012

Density Functional Study of Tetraphenylporphyrin Long-Range Exciton Coupling

Barry Moore, II and Jochen Autschbach*[a]

open_201200020_sm_miscellaneous_information.pdf

The following information is provided in this document: optimized TPP geometry, dimer model geometry, and MM data for idealized dimer geometries. The geometries are given in xyz format for a Cartesian coordinate system (in Å).

TPP Optimized Geometry

78

C -2.429075 2.455719 0.036265
C -2.881253 1.133985 0.129199
C -1.082394 2.851624 -0.051896
C -4.232999 0.693570 0.307541
C -4.235078 -0.679474 0.313626
C -2.884750 -1.125671 0.138065
C -0.672054 4.235202 -0.264731
C 0.685866 4.233842 -0.257709
C 1.090878 2.849137 -0.042108
N -2.104773 0.002644 0.047716
H -1.082801 0.000841 -0.026302
H -5.082795 1.358034 0.442085
H -5.086925 -1.339917 0.454525
N 0.002804 2.024357 0.055183
H -1.339087 5.078531 -0.431976
H 1.356286 5.075674 -0.418924
C -2.436232 -2.449442 0.052202
C -1.090878 -2.849137 -0.042108
C -0.685866 -4.233842 -0.257709
C 0.672054 -4.235202 -0.264731
C 1.082394 -2.851624 -0.051896
N -0.002804 -2.024357 0.055183
H -1.356286 -5.075674 -0.418924
H 1.339087 -5.078531 -0.431976
C 2.436232 2.449442 0.052202
C 4.235078 0.679474 0.313626
C 4.232999 -0.693570 0.307541
C 2.881253 -1.133985 0.129199

N 2.104773 -0.002644 0.047716
H 5.082795 -1.358034 0.442085
C 2.429075 -2.455719 0.036265
C 2.884750 1.125671 0.138065
H 5.086925 1.339917 0.454525
H 1.082801 -0.000841 -0.026302
C 3.480497 3.507900 0.052519
C 4.377530 3.619665 -1.017142
C 3.571450 4.412247 1.117903
C 5.348128 4.621345 -1.023451
C 4.539389 5.416333 1.110600
C 5.429836 5.523495 0.039752
H 4.294790 2.920612 -1.853361
H 2.874512 4.316943 1.954356
H 6.036251 4.705243 -1.867496
H 4.602098 6.115377 1.947483
H 6.186890 6.311007 0.033329
C 3.471824 -3.515634 0.026206
C 4.360042 -3.625548 -1.050873
C 3.572459 -4.420252 1.090392
C 5.331986 -4.625863 -1.065567
C 4.543105 -5.421833 1.075622
C 5.424781 -5.527181 -0.002668
H 4.270511 -2.925210 -1.885381
H 2.882879 -4.325222 1.932958
H 6.014190 -4.707580 -1.914667
H 4.615904 -6.119402 1.912867
H 6.184962 -6.311551 -0.014256
C -3.471824 3.515634 0.026206
C -4.360042 3.625548 -1.050873
C -3.572459 4.420252 1.090392
C -5.331986 4.625863 -1.065567
C -4.543105 5.421833 1.075622
C -5.424781 5.527181 -0.002668
H -4.270511 2.925210 -1.885381
H -2.882879 4.325222 1.932958

H -6.014190 4.707580 -1.914667
H -4.615904 6.119402 1.912867
H -6.184962 6.311551 -0.014256
C -3.480497 -3.507900 0.052519
C -4.377530 -3.619665 -1.017142
C -3.571450 -4.412247 1.117903
C -5.348128 -4.621345 -1.023451
C -4.539389 -5.416333 1.110600
C -5.429836 -5.523495 0.039752
H -4.294790 -2.920612 -1.853361
H -2.874512 -4.316943 1.954356
H -6.036251 -4.705243 -1.867496
H -4.602098 -6.115377 1.947483
H -6.186890 -6.311007 0.033329

TPP Dimer Geometry

156

C -2.1038457364 -9.8401819791 20.0184879814
C -1.0466406185 -9.0927694543 19.4861915316
C -3.3147213687 -9.3110192511 20.4996750474
C 0.2254461095 -9.5873521214 19.0502115972
C 0.9502845608 -8.5261850024 18.5665885248
C 0.1483446323 -7.3458077727 18.6951242119
C -4.4260113751 -10.1441743328 20.9452503653
C -5.3994647710 -9.2911808652 21.3563051294
C -4.8744052006 -7.9449022832 21.1530640398
N -1.0393977656 -7.7356307630 19.2671266641
H -1.7986762372 -7.0991630066 19.5282493390
H 0.5388699021 -10.6254967929 19.1254203097
H 1.9640663176 -8.5391595215 18.1745773219
N -3.6166580433 -7.9805644188 20.6139478802
H -4.4592099078 -11.2314649748 20.9111420503
H -6.3918570378 -9.5375150186 21.7287641312

C 0.4920191496 -6.0465565551 18.3031533112
C -0.2931868768 -4.8980541567 18.5069858696
C 0.0672006033 -3.5852068100 17.9829376056
C -0.9022814450 -2.7303552255 18.4000938695
C -1.8469389770 -3.5312249012 19.1719015621
N -1.4684978834 -4.8461310579 19.2067802223
H 0.9278890684 -3.3658986888 17.3541999984
H -0.9980210691 -1.6687661373 18.1809822183
C -5.5960911938 -6.7835502374 21.4839293589
C -5.8578739515 -4.2636893077 21.6081418079
C -5.1292393662 -3.2019335538 21.1316845737
C -3.9892345120 -3.7224008219 20.4369928718
N -4.0639439125 -5.0918423285 20.5276389331
H -5.3359288146 -2.1430901028 21.2650907151
C -2.9911302242 -2.9888631534 19.7844141274
C -5.1894391988 -5.4702978292 21.2197827249
H -6.7683966716 -4.2296717779 22.2010145271
H -3.3540112719 -5.7382928385 20.1700510299
C -6.9012634386 -6.9486889678 22.1782362652
C -8.0992993552 -6.6265171465 21.5291892570
C -6.9423185310 -7.4387420284 23.4892949801
C -9.3213994799 -6.7933176722 22.1815343402
C -8.1637748901 -7.6081467470 24.1409810907
C -9.3558925972 -7.2846375145 23.4887334376
H -8.0626665876 -6.2553230764 20.5018709930
H -6.0033499763 -7.6819396199 23.9928400880
H -10.2512884341 -6.5456549814 21.6648444599
H -8.1846561199 -7.9879327413 25.1649472065
H -10.3127742191 -7.4172180924 23.9989604743
C -3.1684709274 -1.5127233505 19.7441136645
C -4.1964663826 -0.9409340185 18.9849631999
C -2.3004194433 -0.6790767679 20.4595069284
C -4.3492027461 0.4446669853 18.9349174600
C -2.4529267670 0.7064478774 20.4105448149
C -3.4761155347 1.2714250636 19.6456291067
H -4.8638956643 -1.5950693118 18.4182118349

H -1.5045387623 -1.1313154698 21.0565562486
H -5.1462578538 0.8816331852 18.3292901311
H -1.7711775521 1.3479188834 20.9731795109
H -3.5937990416 2.3566982737 19.6042048237
C -1.9164020140 -11.3152930954 20.0624861809
C -1.8611365517 -12.0575321467 18.8764215353
C -1.7959685370 -11.9775247450 21.2901571387
C -1.6909703080 -13.4414779446 18.9165907823
C -1.6266982826 -13.3615725341 21.3311926247
C -1.5733858833 -14.0964935784 20.1445766555
H -1.9710568412 -11.5383633435 17.9209211853
H -1.8309198246 -11.3928768740 22.2127767596
H -1.6603137558 -14.0122643443 17.9858395824
H -1.5293068241 -13.8681310856 22.2938842460
H -1.4413049436 -15.1805412447 20.1767365616
C 1.7995114882 -5.8869456800 17.6114521253
C 1.9894481475 -6.4082298197 16.3260889088
C 2.8522957586 -5.2085905770 18.2372910860
C 3.2118117145 -6.2486764836 15.6732210040
C 4.0751225614 -5.0495955982 17.5856697966
C 4.2576268716 -5.5692616727 16.3020382998
H 1.1611030781 -6.9271088594 15.8368264566
H 2.7016115897 -4.8108463060 19.2440460232
H 3.3461608314 -6.6498773628 14.6661478914
H 4.8916332022 -4.5236569160 18.0852522996
H 5.2155703744 -5.4454084997 15.7916683869
C -0.0947270000 5.6477040000 -20.9978980000
C -1.0902320000 5.9533280000 -21.9333430000
C -0.0930860000 6.0419080000 -19.6479480000
C -1.0927210000 5.6282490000 -23.3288770000
C -2.2560520000 6.1108650000 -23.8756010000
C -3.0044000000 6.7488400000 -22.8332260000
C 0.9226230000 5.6068800000 -18.6952860000
C 0.6047300000 6.1882430000 -17.5100390000
C -0.5998480000 6.9751200000 -17.7515110000
N -2.2586790000 6.6330850000 -21.6843550000

H -2.5128950000 7.0427250000 -20.7799920000
H -0.2834790000 5.1126790000 -23.8400610000
H -2.5714830000 6.0604020000 -24.9148750000
N -1.0169840000 6.8563550000 -19.0499830000
H 1.7425660000 4.9233300000 -18.9067540000
H 1.1099410000 6.0754620000 -16.5528510000
C -4.2514240000 7.3735200000 -22.9514080000
C -4.9275100000 8.0523310000 -21.9225170000
C -6.2694020000 8.6017450000 -22.0828380000
C -6.5819480000 9.1906990000 -20.8998390000
C -5.4273480000 8.9956370000 -20.0296380000
N -4.4430920000 8.2860880000 -20.6637960000
H -6.8907800000 8.5136290000 -22.9718950000
H -7.5122970000 9.6820710000 -20.6221030000
C -1.2237580000 7.7435950000 -16.7523520000
C -3.0478130000 9.3023630000 -15.9315580000
C -4.2083740000 9.7892260000 -16.4800670000
C -4.3360140000 9.2472480000 -17.8003310000
N -3.2363000000 8.4491420000 -18.0077400000
H -4.9117170000 10.4868910000 -16.0324100000
C -5.3718530000 9.4834750000 -18.7118040000
C -2.4260150000 8.4457910000 -16.8975490000
H -2.6308450000 9.5291260000 -14.9535230000
H -3.0221470000 7.9696050000 -18.8877490000
C -0.5571010000 7.8342990000 -15.4254750000
C -1.1263630000 7.2273160000 -14.2998060000
C 0.6538590000 8.5246120000 -15.2927790000
C -0.4928990000 7.3073990000 -13.0591820000
C 1.2878890000 8.6044290000 -14.0532070000
C 0.7155210000 7.9963000000 -12.9336020000
H -2.0652130000 6.6788580000 -14.4110640000
H 1.0893000000 9.0035120000 -16.1734060000
H -0.9405700000 6.8236300000 -12.1882390000
H 2.2296190000 9.1498930000 -13.9594640000
H 1.2117470000 8.0588590000 -11.9623960000
C -6.4988780000 10.3275830000 -18.2311940000

C -7.3754560000 9.8502020000 -17.2496540000
C -6.6956750000 11.6091290000 -18.7598660000
C -8.4342530000 10.6416120000 -16.8039700000
C -7.7548210000 12.4005940000 -18.3155890000
C -8.6268600000 11.9182110000 -17.3368200000
H -7.2259740000 8.8443480000 -16.8490540000
H -6.0034960000 11.9800810000 -19.5199230000
H -9.1180080000 10.2564400000 -16.0443250000
H -7.8970240000 13.4007010000 -18.7309140000
H -9.4569780000 12.5374880000 -16.9891820000
C 1.0548910000 4.8408170000 -21.4897290000
C 0.8848050000 3.4938190000 -21.8304920000
C 2.3230680000 5.4220250000 -21.6074510000
C 1.9678620000 2.7371780000 -22.2791190000
C 3.4063800000 4.6661130000 -22.0551660000
C 3.2309790000 3.3221100000 -22.3925770000
H -0.1043710000 3.0411300000 -21.7250740000
H 2.4488340000 6.4758050000 -21.3462000000
H 1.8271820000 1.6842940000 -22.5332830000
H 4.3909680000 5.1298670000 -22.1471890000
H 4.0795020000 2.7298960000 -22.7428640000
C -4.9015970000 7.3083180000 -24.2881770000
C -5.4100910000 6.0973250000 -24.7711980000
C -5.0136100000 8.4611760000 -25.0742830000
C -6.0260880000 6.0398340000 -26.0214920000
C -5.6285630000 8.4043700000 -26.3249030000
C -6.1365510000 7.1934400000 -26.8009490000
H -5.3293320000 5.2025410000 -24.1487190000
H -4.6083680000 9.4024700000 -24.6945020000
H -6.4280040000 5.0918570000 -26.3860390000
H -5.7072020000 9.3083410000 -26.9328530000
H -6.6181580000 7.1486420000 -27.7804320000

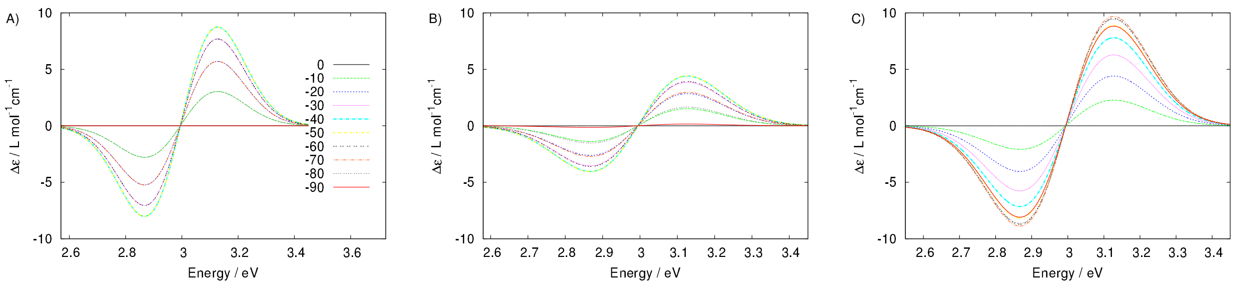

Figure S1: 2x2 matrix method coupling of B_x transitions for three sets of α angles (left to right, see text), with $\beta = 0°$ to $\beta = –90°$.

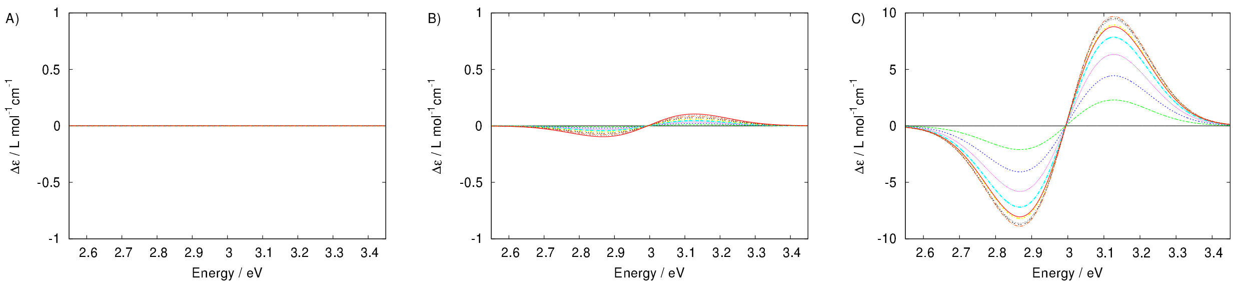

Figure S2: 4x4 matrix method coupling of degenerate $B_{x,y}$ transitions for three sets of α angles (left to right, see text) for $\beta = 0°$ to $\beta = –90°$.

Matrix Method (MM) calculations for an idealized TPP dimer geometry

Consider idealized TPP dimer geometries as discussed in Section 3.3 of the main article, with r_{ab} = 42Å. The system are explored for β = 0 going to $\beta = -90°$, in 10° intervals, for three cases: coupling of B_x only (one per monomer, Figure S1), coupling of degenerate B_x and B_y (Figure S2), and coupling of non-degenerate B_x/B_y transitions (Figure S3). For the degenerate case we set the excitation energy for $B_y = B_x$ and $d_y = d_x$ (vector magnitudes are in the y and x directions, respectively). The resulting CD spectra are plotted for $\alpha_a = \alpha_b$ = 90° (A), α_a = 90° / α_b = 45° (B), and α_a = 135° / α_b = 45° (C) for changing β. The x direction has been shosen to coincide with the transition dipole orientations for $\alpha = 90°$ (see Figure 10 in main article). In Figure S1A, the key is representative of all plots; the printed value represents the β angle. $E_{a/b}$ was set to 3.0 eV for the 2x2 and the degenerate 4x4 cases, while E_b was increased by 0.1 eV for the non-degenerate cases. The $d_{x/y}$ magnitude was set to 3.0 a.u. for all spectra.

In Figure S1 the 2x2 matrix problem yields the predicted CD sign pattern. For $\alpha_a = \alpha_b = 90°$ the plots are symmetric about $\beta = -45°$ due to the $\sin\beta$ term in $R_{1/2}$ (Equation 7a). In Figure S2, the 4x4 matrix problem is more complicated. For $\alpha_a = \alpha_b = 90°$ no net CD intensity is obtained for any value of β because of exact cancellation of the calculated rotatory strengths. In the 4x4 case with $\alpha_a = 90°$ and $\alpha_b = 45°$ the calculated CD has low intensity. When $\alpha_a = 135°$, the CD intensity reaches the same magnitude as the CD obtained from coupling one transition per

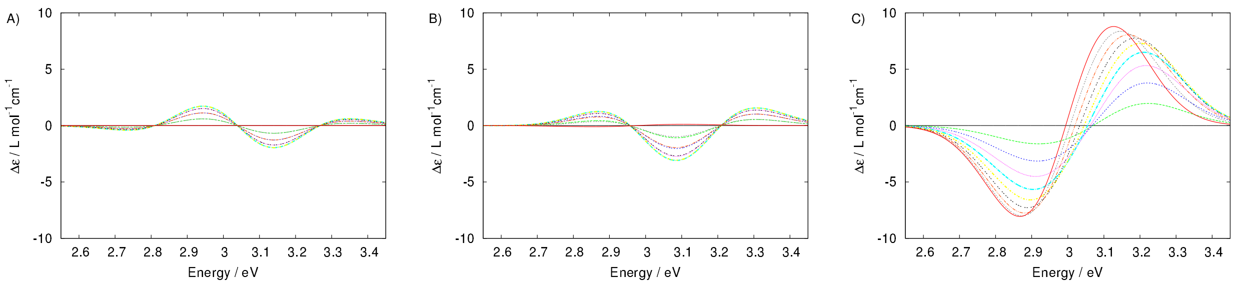

Figure S3: 4x4 matrix method coupling for non-degenerate $B_{x,y}$ transitionsfor three sets of α angles (left to right, see text) for $\beta = 0°$ to $\beta = –90°$.

monomer (Figure S1). When the transitions are non-degenerate the picture becomes comparable to the case observed in the article. When the α terms are 90° the spectrum is split into two weak negative CD couplets centered at 2.8 and 3.3 eV respectively. When $\alpha_b = 45°$ a spectrum emerges where the negative peak at higher energy dominates the spectrum. In system C, with $\alpha_a = 135°$, as β approaches –90° the dipole interaction potential for the B_y transitions becomes very small, and therefore the CD couplet is dominated by interaction of the B_x transitions only, leading to a negative CD couplet centered at 3 eV, with an intensity comparable to that seen in Figures S1 and S2.
